# Ultrastructural and Molecular Characterisation of an *Heterosporis*-Like Microsporidian in Australian Sea Snakes (Hydrophiinae)

**DOI:** 10.1371/journal.pone.0150724

**Published:** 2016-03-23

**Authors:** Amber K. Gillett, Richard Ploeg, Peter J. O’Donoghue, Phoebe A. Chapman, Richard I. Webb, Mark Flint, Paul C. Mills

**Affiliations:** 1 *Vet*-MARTI, School of Veterinary Science, The University of Queensland, Gatton, Queensland, Australia; 2 Australia Zoo Wildlife Hospital, Beerwah, Queensland, Australia; 3 Faculty of Veterinary and Agricultural Sciences, University of Melbourne, Werribee, Victoria, Australia; 4 School of Chemistry and Molecular Biosciences, The University of Queensland, St Lucia, Queensland, Australia; 5 Centre for Microscopy and Microanalysis, The University of Queensland, St Lucia, Queensland, Australia; 6 School of Forest Resources and Conservation, University of Florida, The Florida Aquarium’s Centre for Conservation, Apollo Beach, Florida, United States of America; Illinois Institute of Technology, UNITED STATES

## Abstract

Four sea snakes (two *Hydrophis major*, one *Hydrophis platurus*, one *Hydrophis elegans*) were found washed ashore on different beaches in the Sunshine Coast region and Fraser Island in Queensland, Australia between 2007–2013. Each snake had multiple granulomas and locally extensive regions of pallor evident in the hypaxial and intercostal musculature along the body. Lesions in two individuals were also associated with vertebral and rib fractures. Histological examination revealed granulomas scattered throughout skeletal muscle, subcutaneous adipose tissue and fractured bone. These were composed of dense aggregates of microsporidian spores surrounded by a mantle of macrophages. Sequences (ssrRNA) were obtained from lesions in three sea snakes and all revealed 99% similarity with *Heterosporis anguillarum* from the Japanese eel (*Anguillarum japonica)*. However, ultrastructural characteristics of the organism were not consistent with those of previous descriptions. Electron microscopic examination of skeletal muscle revealed large cysts (not xenomas) bound by walls of fibrillar material (*Heterosporis*-like sporophorocyst walls were not detected). The cysts contained numerous mature microsporidian spores arranged in small clusters, sometimes apparently within sporophorous vesicles. The microspores were monomorphic, oval and measured 2.5–3.0 μm by 1.6–1.8 μm. They contained isofilar polar filaments with 11 (infrequently 9–12) coils arranged in two ranks. This is the first published report of a microsporidian infection in hydrophiid sea snakes. This discovery shows microsporidia with molecular affinities to *Heterosporis anguillarum* but ultrastructural characters most consistent with the genus *Pleistophora* (but no hitherto described species). Further studies are required to determine whether the microsporidian presented here belongs to the genus *Heterosporis*, or to a polymorphic species group as suggested by the recognition of a robust *Pleistophora/Heterosporis* clade by molecular studies. The gross and histological pathology associated with these infections are described.

## Introduction

Microsporidia are opportunistic intracellular spore-forming pathogens [1]. Although suggested as sharing a common ancestor with fungi [2–4], microsporidia do not resemble fungi morphologically, nor are they considered true fungi [5]. Recent taxonomic classification positions the phylum Microsporidia alongside the Aphelida and Cryptomycota within the superphylum Opisthosporidia [6]. The phylum Microsporidia contains more than 1,300 species belonging to over 180 genera [4–7] infecting many hosts ranging from invertebrates to humans. Classification into groups or clades is often largely based on habitat and host species [8]. Infections are often systemic and tissue tropism is exhibited for many species [9], with the latter often being used to assist with phylogenetic classification. Ecological niches are displayed among microsporidian clades and infections are recognized in terrestrial, freshwater and marine hosts although almost half the known genera of microsporidians infect aquatic hosts [8]. Known as a cause of disease in invertebrates such as silkworms and bees, as well as fish, crustaceans and eels [10–13], outbreaks of infection may result in significant economic losses. Of greatest impact in aquaculture facilities, microsporidian infections result in significant muscle damage, growth deformities, reduced productivity as well as unsightly flesh, most notably in farmed fish, crustaceans and eels [12, 13]. Immunocompromised humans are also susceptible to infections of the gastrointestinal tract [14] and muscle [15] and infections in transplant recipients [16] have been reported in the corneas of otherwise healthy individuals [1].

Traditionally, the classification of microsporidia was based on morphology alone, but this has been challenged more recently with the application of molecular genetics [8, 17]. Comparative analysis of small subunit ribosomal RNA (ssrRNA) sequence data challenges the existing taxonomy [18]. In addition to a demonstrated tissue tropism, important morphological characteristics of these organisms include: the size of mature spores; the arrangement and the number of coils of the polar tube; nuclear arrangement; presence or absence of a sporophorous vesicle (SPV) and sporophorocyst (SPC); monokaryotic or diplokaryotic status of merogonous and sporogonous developmental stages; and the organisms capacity to induce xenoma formation. Morphological plasticity among microsporidia is a recently recognized phenomena [17] and can lead to conflicting results in speciation when phylogenetic and ultrastructural characteristics are used. The use of ssrRNA-based phylogenetics has identified ‘clades’ within the phylum Microsporidia [9] and it has even been proposed that the classes Terresporidia, Aquasporidia and Marinosporidia be recognized based on host habitat colonization [8]. The use of ssrRNA-based phylogenetics is currently proposed as the main discriminator for relatedness amongst microsporidia [9], yet its use as a sole method for taxonomic classification is limiting and often confined to sequence data from one region of the genome. Thus, the most robust method for describing novel microsporidia is achieved with the integration of a range of features including host type, ecology, pathology, ultrastructural morphology and phylogenetics [8, 9].

Documentation of microsporidians in reptiles is limited and only a few publications are available describing macrospore and microspore morphology and sequence data [19, 20]. Published reports in reptiles include infections with *Heterosporis anguillarum* (host species: *Thamnophis sirtalis*) [20, 21], *Pleistophora atretii* (host species: *Atretium schistosum gunther*) [22], *Encephalitozoon lacertae* (host species: *Podarcis muralis* and *Mabuya perrotetii*) [19, 23], *Glugea danilewski* (host species: *Natrix natrix*) [24], *Pleistophora* sp. (host species: *Sphenodon punctatus*) [25] and an unidentified microsporidian species (host species: *Pogona vitticeps*) [26]. Infections are limited to skeletal muscle or the gastrointestinal tract but may also be systemic. A brief report of microsporidia in three Australian reptile species exists in the Taronga Zoo Wildlife Pathology Registry [27] from 1992. The host species included a central knob-tailed gecko (*Nephrurus amyae*), water dragon (*Intellagama lesueurii*) and a yellow bellied sea snake (*Hydrophis platurus*), but although Hartley and Reece (unpublished) provided a short description of refractile, acid-fast staining organisms surrounded by an inflammatory reaction, there was no further morphological description or suggested identification of the organisms. These organisms induced widespread myonecrosis and within the ovary of the gecko, some ova were “replaced by masses of microsporidia within macrophages” [27]. These reports were later cited by Ladds (p269) [28] however no further information on pathogenicity, morphology or identification was provided.

Marine snakes from the subfamily Hydrophiinae (true sea snakes) live an entirely marine existence, as opposed to their counterparts the Laticaudinae (sea kraits) that spend various amounts of time on land. More than 60 species of hydrophiids are recognized, with approximately 30 species permanently inhabiting Australian waters [29]. Considerable variation in distribution exists, as well as microhabitat use, feeding methods and prey species selection among sympatric species. The large majority of hydrophiid sea snakes feed on marine eels and bony fishes, however, a small number of species with a reduced venom apparatus feed exclusively on fish eggs (*Emydocephalus annulatus* and *Aipysurus eydouxii*) [30, 31]. Sea snakes occupy differing habitat niches with some species existing in shallow coral reefs (e.g. *Aipysurus* sp.), some in estuarine and soft sediment habitats (e.g. *Hydrophis elegans* and *Hydrophis major*) and others living an entirely pelagic existence (e.g. *Hydrophis platurus*). Few investigations into disease and mortality in sea snakes have been done other than analyses relating to epibiota [32], the effects of trawling on population decline [33], and a recent publication by Gillett *et al*. [34] on sea snake assessment and veterinary examination.

This is the first published report of microsporidian infections in hydrophiid sea snakes and describes morphological and molecular sequence data (ssrRNA) as well as the associated gross and histological pathology.

## Materials and Methods

### Sample collection

Eighty three sea snakes (Hydrophiinae) collected following stranding between Fraser Island (-24.709138, 153.257189), Queensland, Australia and the Gold Coast (-28.164607, 153.526735), Queensland, Australia were necropsied at Australia Zoo Wildlife Hospital (AZWH) from 2007 to 2013. Snakes were either found deceased on the beach, died shortly after collection, or were euthanased due to untreatable conditions found during veterinary examination, as per Gillett *et al*. 2014 [34]. All work was conducted under the AZWH rehabilitation permit (as snakes were rescued wildlife) and ethics permit issued by the University of Queensland ethics committee (SVS/442/10/AUSZOO/VET-MARTI/QDERM).

During gross necropsy, isolated granulomas were identified in the hypaxial and intercostal musculature at varying locations along the body in 4/83 snakes. Three of these snakes were collected from separate locations on the Sunshine Coast region, Queensland and the fourth was collected from Fraser Island, Queensland. All affected snakes were collected in separate years, and at differing times of the year (Table 1). All snakes were identified to species level, given a body condition score (as per Gillett *et al*. 2014), weighed and a snout to vent length (SVL) was recorded. In 4/4 snakes, small pieces of the affected tissue were collected in 10% neutral buffered formalin and subsequently processed for histopathology. In 3/4 snakes (HM2, HM1 and HE1), additional samples of the affected tissue were frozen and stored at -80°C. The formalin fixed samples were paraffin-embedded, sectioned (3 to 5 μm) and stained with haematoxylin and eosin. Subsequently the formalin-fixed, paraffin-embedded tissue was utilised for transmission electron microscopy.

**Table 1:** Case details for sea snakes affected by microsporidia.

ID	Species	Stranding date	Stranding location	Latitude	Longitude	Weight (grams)	SVL (cm)	Body condition	Disease/Injury status
HP1	*Hydrophis platurus*	5/01/2007	Currimundi Lake, Sunshine Coast, QLD	-26.7657	153.1369	270	NA	NA	Trauma—Spinal fracture and osteomyelitis
HM1	*Hydrophis major*	26/07/2011	Dundubara, Fraser Island, QLD	-25.1575	153.1440	890	130	Good	Trauma—Rib fractures and osteomyelitis
HM2	*Hydrophis major*	28/11/2012	Mooloolabah Beach, Sunshine Coast, QLD	-26.6827	153.1248	430	120	Poor	Neoplasia–disseminated adenocarcinoma
HE1	*Hydrophis elegans*	2/08/2013	Marcoola Beach, Sunshine Coast, QLD	-26.5820	153.0979	450	133.7	Emaciated	Neoplasia–disseminated adenocarcinoma

HP1, *Hydrophis platurus* 1; HM1, *Hydrophis major* 1; HM2, *Hydrophis major* 2; HE1, *Hydrophis elegans* 1

### Molecular characterisation and phylogeny

A 25 mg piece of fresh-frozen tissue from each of the three individuals (HM1, HM2 and HE1) was processed for DNA extraction using a DNEasy Blood and Tissue kit (Qiagen, Chadstone, Victoria), as per manufacturer directions. ssrRNA gene sequences were amplified by PCR using the universal microsporidial primers 18f (5′-CAC CAG GTT GAT TCT GCC-3′) and 1492r (5′-GGT TAC CTT GTT ACG ACT T-3`) for amplifying unknown rRNA genes in novel microsporidia species [35]. PCR was performed in a 25 μL reaction volume, comprising 6 μL of DNA, 2.5 μL 10x PCR buffer (Qiagen, Chadstone, Victoria), 4 μL of dNTP at 1.25 mM (Qiagen, Chadstone, Victoria), 2.5 μL each of 18f and 1492r primer at 10 mM, 1.25 units of HotStarTaq (Qiagen, Chadstone, Victoria) and the remaining volume made of nuclease-free water. A negative control sample containing nuclease free water in place of DNA was run in parallel. Amplification was carried out in a Biorad C1000 thermal cycler. After initial denaturation for 4 minutes at 94°C, 35 cycles were completed of the following: denaturation at 94°C for 50 seconds, annealing at 56°C for 50 seconds and extension at 72°C for 80 seconds. A final extension of 7 minutes at 72°C was completed before holding at 4°C. The resulting PCR amplification product was analysed by gel electrophoresis prior to sequencing. The PCR product was then purified and sequenced by the Animal Genetics Laboratory (AGL) within the School of Veterinary Science, The University of Queensland. Chromatographs were read and analysed using the software program Finch TV v1.4.0 (Geospiza Inc., Seattle, WA) and contigs assembled by combining corresponding forward and reverse sequences. Sea snake sequences were aligned using the ClustalW accessory application within BioEdit c1.0.9.0 [36]. A BLAST search was completed to determine similarity with other sequences in GenBank. All sequences with greater than 92% sequence similarity and greater than 90% query cover were included in phylogenetic analysis. To assist with determining identity and phylogenetic placement, at least one representative from each major microsporidian group (as per Vossbrinck, 2005) was also included in phylogenetic analysis. Sequences selected represented species from freshwater, terrestrial and marine hosts and from the classes Aquasporidia, Marinosporidia and Terresporidia. Non-microsporidian outgroups for phylogenetic analysis included a representative from the neighbouring Aphelidia, Cryptomycota, Chytridiomycota and Zygomycota phyla [6]. Partial ssRNA sequences were used to generate phylogeny. All sequences were aligned using Muscle [37] to a final alignment length of 1920 positions (the inclusion of non-microsporidian outgroups resulted in increased dataset length—Data in S1 Dataset). The maximum likelihood tree was constructed using PhyML [38] with 1000 bootstrap replications. Bootstrap values were expressed as percentages and only values above 50 were shown in the final tree. The TIM3 + G + I substitution model was specified based the results of a jModelTest 2.0 analysis of the alignment. MrBayes 3.2.4 [39] was used to construct a Bayesian inference tree using 3 million generations, sample frequency of 100 and a burn-in value of 10%. As TIM3 is not supported by MrBayes, the GTR + G + I model was specified as the closest over-parameterised model [40]. Convergence and burn-in values were assessed using Tracer [41]. Only posterior probabilities above 50 were shown in the final tree. Sea snake microsporidia sequences were submitted to GenBank with the accession numbers KT380106, KT380107 and KT380108.

### Transmission electron microscopy

Only formalin fixed tissues were available for transmission electron microscopy (tEM). Tissues were re-fixed with 2.5% glutaraldehyde in 0.1 M cacodylate buffer. All processing was done with the assistance of a Pelco Biowave microwave oven (Ted Pella, Redding, USA). The samples were then washed with 0.1 M cacodylate buffer, and post fixed with 1% osmium tetroxide in 0.1 M cacodylate buffer. They were further washed with ultra-high quality water then dehydrated through a graded acetone series and infiltrated with Epon resin and polymerised at 60°C for 2 days. Ultramicrotomy was performed on a Leica Ultracut UC6 ultramicrotome and sections of 50 nm thickness were mounted on copper grids and stained with 5% uranyl acetate in 50% methanol, followed by Reynolds lead citrate. The sections were viewed in a JEOL 1010 transmission electron microscope operated at 80 kV and images were collected on an Olympus Soft Imaging Veleta digital camera.

## Results

### Sea snake species

Two snakes were identified as *Hydrophis major*, one as *Hydrophis platurus*, and one as *Hydrophis elegans*. All snakes were deemed mature based on snout to vent length (SVL) and weight [34], and all were recovered from different beach locations (Table 1).

### Gross pathology

**Fig 1:**
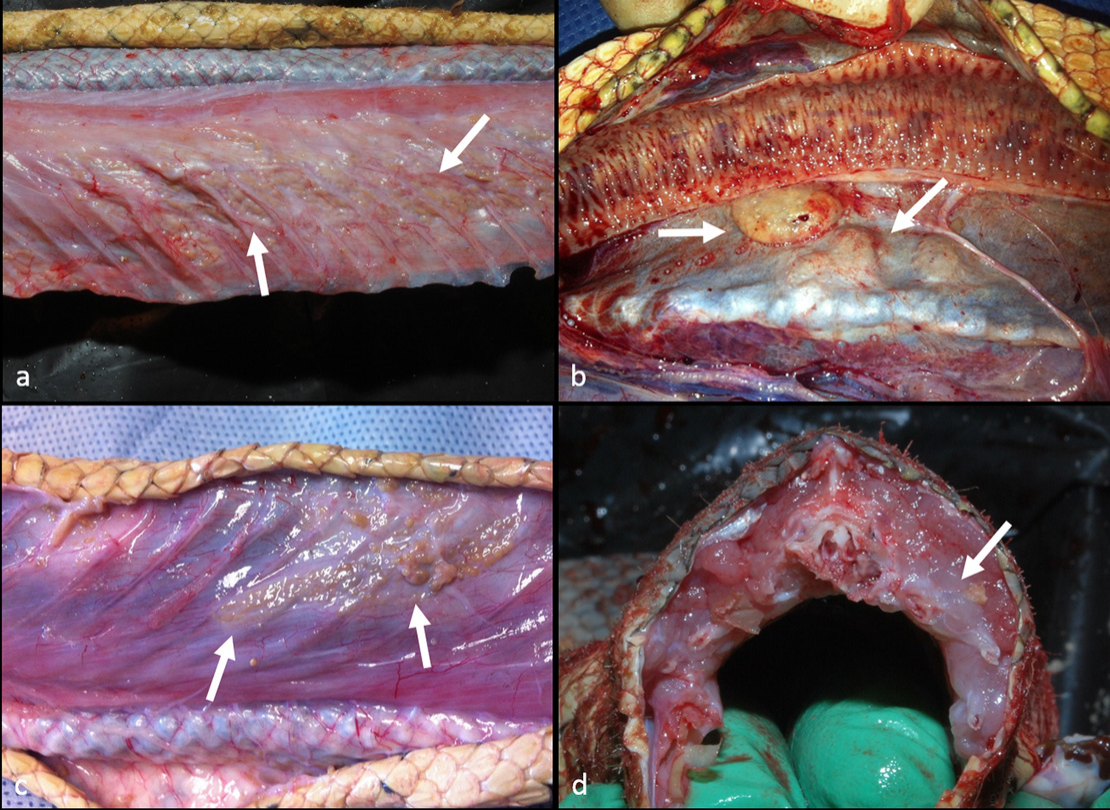
Gross appearance of axial muscles in hydrophiid sea snakes bearing numerous microsporidian granulomas. a) Multiple granulomas (arrows) containing microsporidian parasites associated with costal fractures in *Hydrophis major* (HM1). b) Multiple granulomas (arrows) in the hypaxial muscles adjacent to the spine in *Hydrophis elegans* (HE1). c) Multiple granulomas (arrows) visible in the intercostal muscles of *Hydrophis major* (HM2). d) Locally extensive regions of pallor (arrows) in the hypaxial and intercostal muscles of *Hydrophis major* (HM1).

Two animals had evidence of trauma with a vertebral fracture observed in HP1 and multiple costal fractures in HM1. The two remaining snakes (HM2 & HE1) had multiple, variably-sized, off-white nodules scattered throughout the liver and spleno-pancreas which were histologically identified as adenocarcinomas. Scattered throughout the hypaxial and intercostal musculature were multifocal to coalescing aggregates of off-white to tan tissue (granulomas) often manifesting as locally extensive regions of discoloration. These areas appeared to be concentrated around the spinal injury in HP1 and closely associated with costal fractures in HM1 (Fig 1A). In HM2 and HE1 the granulomas were not associated with any visible trauma and were confined to the thoracolumbar region (Fig 1B & 1C). Locally extensive regions of pallor were evident throughout the muscle adjacent the granulomas (Fig 1D).

### Histopathology

**Fig 2:**
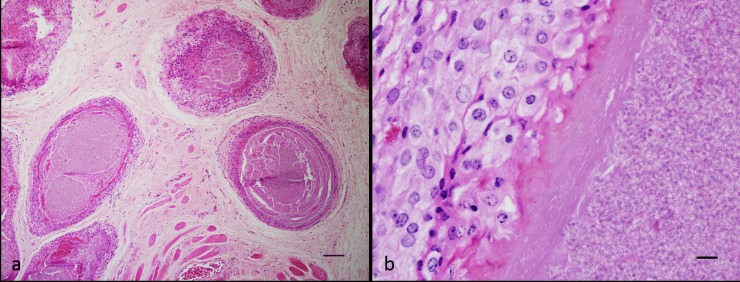
Histopathology of sea snake microsporidia a) Dense aggregates of microsporidian spores scattered throughout skeletal muscle and subcutaneous adipose tissue. Snake HM2. Scale bar 100 μm b) Spore aggregates were surrounded by a mantle of macrophages and enclosed within a delicate fibrous capsule. Snake HM2. Scale bar 10 μm.

Disrupting the skeletal muscle and at times bone, as well as being scattered throughout the adjacent subcutaneous adipose tissue (Fig 2A), were multiple, at times coalescing, dense aggregates of microsporidian spores typically surrounded by a thin mantle of macrophages and further enclosed by a delicate fibrous capsule (Fig 2B). The noted spores were round to ovoid and measured 2.0–2.6 x 1.0–1.5 μm. These had thin refractile walls and on occasion a basophilic crescent-shaped structure was apparent at one pole. The intervening tissue was variably oedematous and contained scattered heterophils and macrophages.

### Transmission electron microscopy

**Fig 3:**
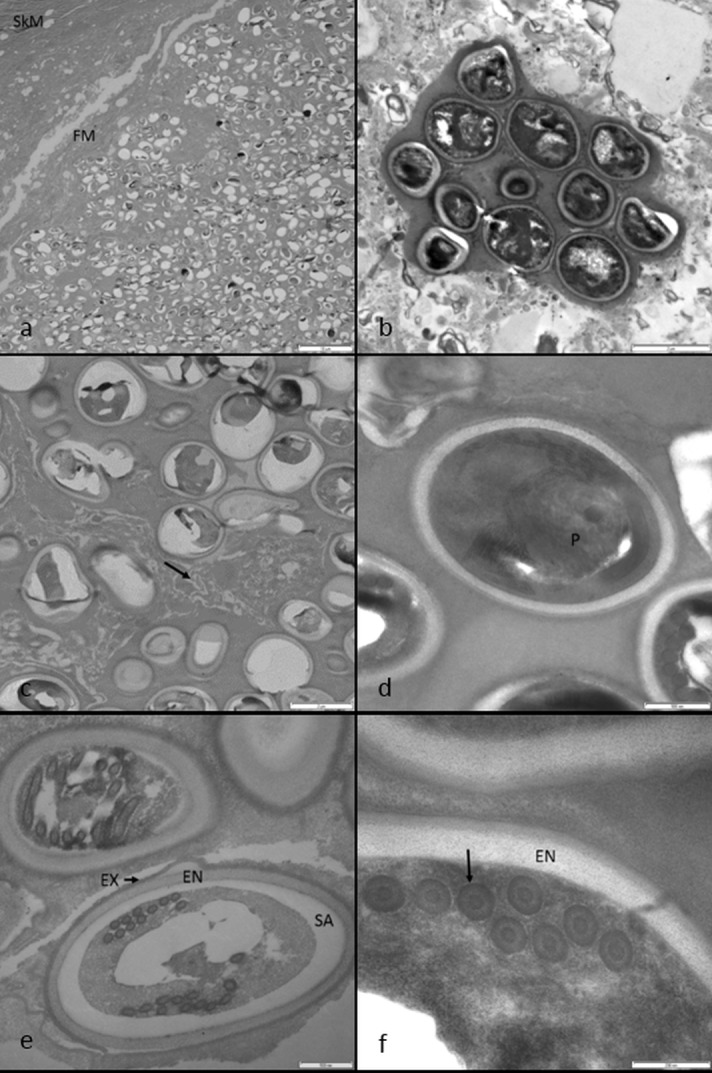
Transmission electron micrographs of sea snake microsporidia a) Cyst containing hundreds of mature microsporidian spores bound by a wall of fibrillar material (FM) in the skeletal musculature (SkM). b) Spores embedded in dense ground substance and apparently surrounded by sporophorous vesicle membranes were occasionally observed. c) Spores were usually embedded in heterogenous ground substance with occasional fibrillar/membranous patches (arrow). d) An irregularly lamellated polaroplast (P) was occasionally seen in sections of mature spores. e) The spore wall consisted of a thin electron-dense exospore wall (EX) with a roughened surface lacking distinct surface projections or protrusions, an inner thick electron-lucent endospore wall (EN) often overlying a shrinkage artifact (SA) separating it from the spore cytoplasmic contents. f) Section through the periphery of a mature spore showing internal cross-sectional detail of isofilar polar filaments arranged in two loose ranks (arrow).

Transmission electron micrographs showed occasional large cysts containing hundreds of refractile microsporidian spores in the skeletal muscle, bound by a wall of fibrillar material (FM) (Fig 3A). Most spores were scattered loosely throughout the cyst, but occasionally clusters of up to 30 spores were embedded in dense homogenous ground substance (Fig 3B), some clusters were apparently bound by a sporophorous vesicle (pansporoblast) membrane. The majority of spores, however, were strewn irregularly throughout the cyst supported by less-dense heterogenous ground substance interspersed with discontinuous aggregates of fibrillar or membranous material (Fig 3C). The spores poorly infiltrated with resin resulting in shrinkage artifacts and holes were evident in many sections. It was difficult to determine if the spores were monokaryotic or diplokaryotic, but where artifacts were not so severe, spores appeared monokaryotic. Many spores contained a posterior vacuole, and an anterior irregularly lamellated polaroplast was occasionally seen (Fig 3D). All spores present were mature in appearance (with well-developed spore walls and polar tubes) and no immature or developing merogonic or sporogonic stages were observed. Mature spores were monomorphic, oval in longitudinal section and measured 2.5–3.0 μm long by 1.6–1.8 μm wide. Spores appeared to have an outer thin electron-dense exospore wall 0.04–0.05 μm wide with a roughened surface lacking any distinct projections or protruding elements (Fig 3E & 3F). An inner thick electron-lucent endospore wall was visible measuring 0.10–0.14 μm and this was often overlaying a shrinkage artifact separating it from the spore cytoplasmic contents (Fig 3E). The spores contained well developed polar filaments, which appeared isofilar and round-oval in cross-section measuring 0.1–0.11 μm long by 0.08 μm wide (Fig 3F). Sections through the polar filaments were located in two ranks lateral to the longitudinal axis suggesting an arrangement of 11 coils (infrequently 9–12).

### Molecular analysis

**Fig 4:**
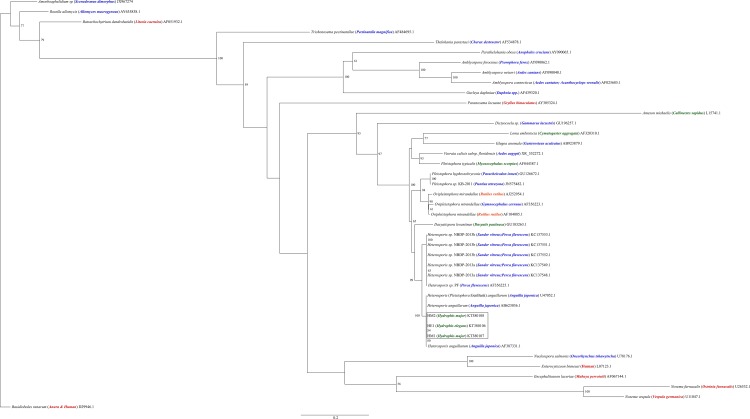
Maximum likelihood analysis of the relationships of sea snake microsporidia (HM1, HM2 and HE1) and representatives of each major microsporidian group based on the ssrRNA gene. Phylogenetic tree constructed using PhyML from 41 sequences using TIM3 + G + I substitution model. Bootstrap values are listed as percentages generated from 1000 iterations. Bootstrap values were expressed as percentages and only values above 50 are shown. GenBank accession numbers are listed after species and host species names. Host species are denoted in parentheses. Color denotes predominant host habitat type (Red; terrestrial, Green; marine, Blue; freshwater, Orange; brackish). Scale bar indicates the number of nucleotide substitutions per site.

**Fig 5:**
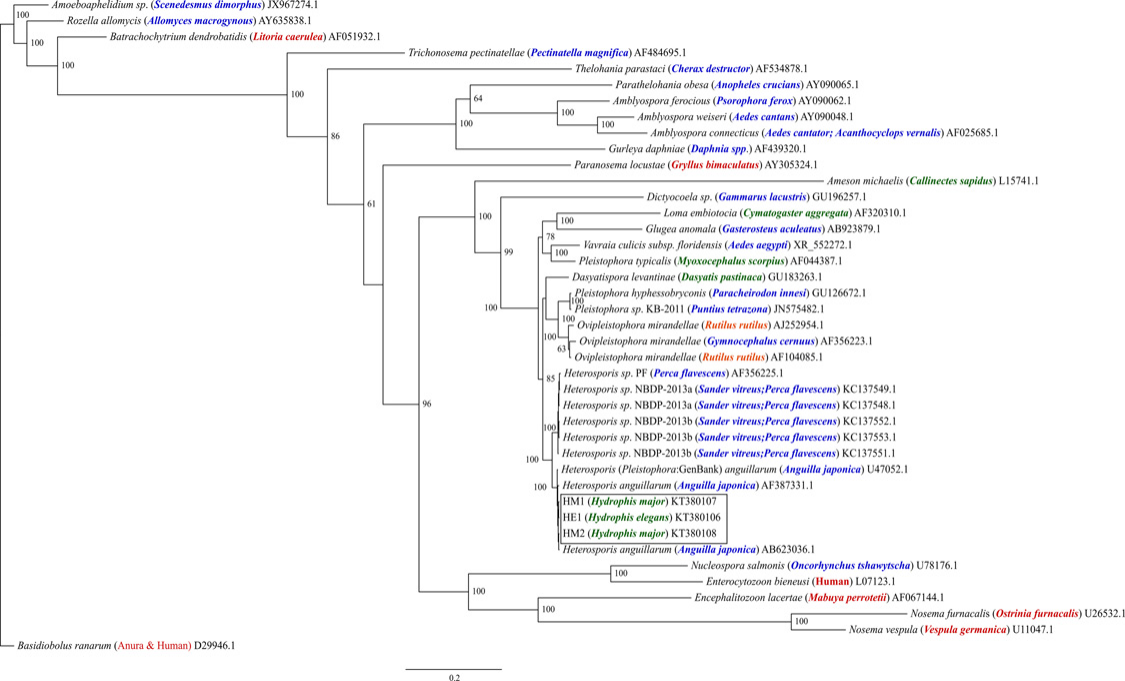
Bayesian analysis of the relationships of sea snake microsporidia (HM1, HM2 and HE1) and representatives of each major microsporidian group based on the ssrRNA gene. PhyloBayes tree topology was constructed from 41 sequences using the GTR + G + I model. Bayesian posterior probabilities are listed as percentages and only values above 50 are shown. GenBank accession numbers are listed after species and host species names. Host species are denoted in parentheses. Color denotes predominant host habitat type (Red; terrestrial, Green; marine, Blue; freshwater, Orange; brackish). Scale bar indicates the number of nucleotide substitutions per site.

Three sequences were amplified from the DNA of spores present in cysts in skeletal muscles from three sea snakes. Comparison of sequences from all three samples revealed 100% similarity except that HM2 had a single degenerate base symbol ‘W’ substituted where HM1 and HE2 had an adenine base. A BLAST search found that sea snake isolates shared 99% sequence identity with *Heterosporis anguillarum* (AB623036.1 & AF387331.1 and U47052.1 [Listed in GenBank as *Pleistophora anguillarum*]) isolated from fresh water eels (*Anguilla japonica*) in Japan and Taiwan. Maximum likelihood and Bayesian inference analyses packages produced trees with very similar topology (Fig 4 & Fig 5). Sea snake isolates formed a well-supported clade with the three freshwater eel isolates. A sister clade was formed by *Heterosporis* spp. isolated from freshwater fish from the USA. The Heterospora clades were included in a broader clade that incorporated *Pleistophora* and *Ovipleistophora* species from brackish and freshwater hosts, *Dasyatispora levantinae* from a stingray (*Dasyatis pastinaca*), *Loma embiotocia* and *Glugea anomala* from marine and freshwater fish and *Vavraia culicis* from mosquitoes. A sister clade with species infecting predominantly terrestrial hosts is represented, and includes *Enterocytozoon bieneusi infecting* humans. Clades affecting predominantly freshwater hosts occupy a more basal position in the tree.

## Discussion

To our knowledge, this is the first report describing microsporidian infections in marine snakes. This manuscript details the gross and microscopic pathology within the host, spore morphology and the phylogenetic relationships with known microsporidian species.

The pathological changes in the axial muscles reported here are consistent with those induced by microsporidia infecting marine fish and marine invertebrates [42, 43]. Although the direct impact of infection could not be assessed in these cases, the involvement of the axial muscles could have been a significant impediment to locomotion in these animals given the crucial role this musculature plays in instigating lateral undulatory movements [44]. Any compromise in the swimming ability of sea snakes could result in an increased risk of predation, reduced feeding success and increased likelihood of anthropogenic injury (e.g. boat strike, trawling) or stranding. Two of the four snakes in this study had evidence of traumatic injury and three had suboptimal body condition (optimal condition described by Gillett *et al*. [34]) which could imply that the presence of microsporidial infection may negatively impact sea snake hosts. It is unlikely that the infections were related to the neoplasms detected in snakes HM2 and HE1 however, immunosuppression as a result of cachexia may have potentiated the risk of infection [45]. The route of infection is unknown, and difficult to establish without a definitive classification, but possibilities include ingestion of spores in infected prey or the colonization of the open wounds noted in the traumatized snakes.

Genera of microsporidia reported to infect the muscle of marine hosts include: *Ameson* in decapods [13], *Pleistophora*, *Heterosporis*, *Kabatana*, *Myosporidium*, *Tetramicra*, *Glugea* and *Microsporidium* in fishes (host order: Anguilliformes, Perciformes) [8] and *Dasyatispora* in stingrays [9], but no species are known to affect marine reptiles. Collectively, the ultrastructural characteristics of the microsporidian parasites found in sea snakes did not conform to those of any previously described species. Morphological characteristics of the microsporidians detected in sea snakes have been compared in Table 2 against selected microsporidian species in fishes, eels, snakes and lizards. The species chosen for comparison infect both freshwater and marine hosts. Comparisons were made with non-xenoma-forming species of *Pleistophora*, *Dasyatispora*, *Ovipleistophora* (all three genera forming sporophorous vesicles), *Kabatana* (without sporophorous vesicles) and *Heterosporis* (forming sporophorocysts) as well as intranuclear species of *Nucleospora*, and xenoma-forming species of *Glugea*, *Loma*, *Myosporidium*, *Tetramicra* and *Potaspora*. Sea snake microsporidia did not form xenomas like *Myosporidium*, *Tetramicra* and *Glugea*; they did not form SPC’s like *Heterosporis*; but appeared to form SPV’s like *Pleistophora* and *Dasyatispora* (but unlike *Kabatana*). Spore shape, size and polar filament number and arrangement were unlike any previously described in these genera, but were most similar to that of an *Ameson* sp. that infects the muscles of crabs, often associated with significant muscle necrosis [13, 69]. However, *Ameson* spp. characteristically have an exospore wall with thin hair-like appendages and diplokaryotic mature spores which were not present in the sea snake microsporidia. *Pleistophora* spp. have been found in ‘cysts’ in the muscles of fish [55, 70] and species such as *Pleistophora hyphessobryconis* show broad host specificity and affect a range of freshwater fish species [55]. The microspores formed by several piscine *Pleistophora* spp. were roughly comparable in size to those detected in this study (though most being 20–50% larger), but they usually contained many more coils of their polar tubes (17–34 cf. 10–12 detected in this study) (11). The unique combination of ultrastructural characters displayed by the microsporidian in sea snakes may suggest tentative placement, on morphological grounds, in the genus *Pleistophora*.

**Table 2:** Morphological characteristics of sea snake microsporidia compared with selected microsporidia in marine and freshwater hosts.

Microsporidian species	Host species	Affected Tissue	Xenoma	SPC	SPV	Macro-spores		Micro-spores	
						Size (mm)	Polar tube coils	Size (mm)	Polar tube coils
*Heterosporis-like microsporidian*. (current publication)	*Hydrophis major*, *H*. *platurus*, *H*. *elegans*	Muscle	Absent	Absent	Present	nr	-	2.5–3.0 x 1.6–1.8	9–12 (2 ranks)
*Plistophora atretii* [22]	*Altretium schistosum*	Muscle	Absent	Absent	Present	nr	-	4.8–5.4 x 1.8–2.5	nr
*Heterosporis anguillarum* [20]	*Thamnophis sirtalis*	Muscle	Absent	nr	nr	5.3–6.8 x 2.0–4.0	29–42 (3–5 ranks)	2.7–3.5 x 1.8–2.4	8–11 (1 rank)
*Heterosporis anguillarum* [11, 12, 46–48] (syn. *Plistophora anguillarum*)	*Anguilla japonica*	Muscle	Absent	Present	Present	6.7–9.0 x 3.3–5.3	33–46 (1–3 ranks)	2.8–5.0 x 2.0–2.9	nr
*Heterosporis sunderlandae* [49]	*Perca flavescens*, *Esox lucius*, *Sander vitreus*	Muscle	Absent	Present	Present	4.8–6.3 x 3.2–3.6	18–21 (1 rank)	nr	-
*Heterosporis saurida* [50]	*Saurida undosquamis*	Muscle	Absent	Present	Present	5.0–6.0 x 3.0–3.8	20–21	3.0–3.8 x 1.5–2.5	5–6 (1 rank)
*Ovipleistophora mirandellae* [11, 51–53] (syn. *Pleistophora mirandellae*, *P*. *longifilis*, *P*. *elegans*, *P*. *oolytica)*	*Alburnus alburnus*, *Rutilus rutilus*, *Leuciscus cephalus*, *Gobio gobio*, *Gymnocephalus cernuus*, *	Ovary, testes	Absent	Absent	Present	7.3–12.0 x 3.5–6.4	nr	3.0–7.5 x 1.5–4.0	nr
*Pleistophora hyphessobryconis* [11, 53–56]	*Paracheirodon inessi*, *Hemigrammus erythrozonus*, *Phoxinus phoxinus*, *Carassius auratus*, *Danio rerio*, *	Muscle	Absent	Absent	Present	6.5–7.0 x 4.0	34	4.0–6.0 x 2.0–3.3	34 (3 ranks)
*Pleistophora typicalis* [11, 53, 57, 58]	*Myoxocephalus (Coyyus) scorpius*, *Cottus bubalis*, *Blennius pholis*, *Gasterosteus pungitius*	Muscle	Absent	Absent	Present	6.3–8.3 x 3.0–3.3	33–39 (3 ranks)	3.0–5.6 x 1.5–3.0	10–22 (1–3 ranks)
*Dasyatispora levantinae* [59]	*Dasyatis pastinaca*	Muscle	Absent	Absent	Present	nr	-	3.8–4.3 x 2.6–2.8	9–12 (2 ranks)
*Nucleospora salmonis* [11, 60, 61] (syn. *Enterocytozoon salmonis*)	*Oncorhynchus tshawytscha*, *O*. *mykiss*	Hemoblast nuclei	Absent	Absent	Absent	nr	-	2.0 x 1.0	8–12
*Kabatana newberryi* [62]	*Eucyclogobius newberryi*	Muscle	Absent	Absent	Absent	nr	-	2.8 x 1.9	9–10 (1–2 ranks)
*Kabatana rondoni* [63]	*Gymnorhamphichthys rondoni*	Muscle	Absent	Absent	Absent	nr	-	4.25 x 2.37	8–10 (2 ranks)
*Glugea anomola* [11, 53]	*Gasterosteus aculeatus*, P*ungitius puingitius*, *P*. *platygaster*, *Gobius minutus*	Connective tissue	Present	Absent	Present	nr	-	3.0–6.0 x 1.5–2.7	Isofilar. (1 rank)
*Glugea vincentiae* [64]	*Vincentia conspersa*	Subcutaneous tissue	Present	Absent	Present	7.5–12.0 x 2.0–4.0	12–14 (1–3 ranks)	4.5–6.0 x 2.0–2.7	12–14 (1–3 ranks)
*Loma embiotocia* [11, 65]	*Cymatogaster aggregata*	Gills	Present	Absent	Present	nr	-	4.0–5.0 x 2.0–3.0	14–18
*Myosporidium merluccius* [66]	*Merluccius capensis/paradoxus*	Muscle	Present	Absent	Present	nr	-	2.5–3.3 x 1.8–2.1	Anisofilar, 11–12 (1–2 ranks)
*Tetramicra brevifilum* [11, 67]	*Scophtalmus maximus*, *Lophius budegassa*	Connective tissue & muscle	Present	Absent	Absent	nr	-	3.7–4.8 x 2.0–2.7	3–4
*Potaspora aequidens* [68]	*Aequidens plagiozonatus*	Muscle	Present	Absent	Absent	nr	-	3.4 x 1.9	8–9

nr, not recorded

*, Other species recorded but not published here.

Phylogenetic analysis of the ssrRNA of the microsporidian found in the tissues of three of the hydrophiid sea snakes investigated here revealed high (99%) sequence similarity to *Heterosporis anguillarum* isolated from freshwater Japanese eels (*A*. *japonica*). If used as the sole method of identification, this may justify the placement of the sea snake microsporidian within the genus *Heterosporis*, perhaps even as the species *H*. *anguillarum*, or at least one within the *Pleistophora/Heterosporis* clade. However, despite molecular affinities with the *Heterosporis* genus, there were distinct differences in cyst and spore morphology between the current organism and members of this clade that cannot be overlooked. Furthermore, sufficient morphological characteristics were appreciable to indicate that this parasite did not completely conform to any current microsporidian species description. In eels (*Anguilla japonica*), *H*. *anguillarum* has been reported to form macrospores measuring 6.7–9.0 x 3.3–5.3 μm with 33–46 polar tube coils and microspores 2.8–5.0 x 2.0–2.9 μm with 33–46 polar tube coils (when recorded) [48]. In garter snakes (*Thamnophis sirtalis*), *H*. *anguillarum* was reported to form macrospores measuring 5.3–6.8 x 2.0–4.0 μm with 29–42 coils and microspores measuring 2.7–3.5 x 1.8–2.4 μm with 8–11 coils (20). While the microspores found in the sea snakes closely resembled those found in garter snakes, there was no evidence for the presence of macrospores, but more importantly, a sporophorocyst encompassing all developmental stages was not present. The results of the morphotypic and molecular characterization studies are therefore not congruent. The absence of pre-spore developmental stages and problems with tissue infiltration precluded a more comprehensive morphological assessment required for accurate species identification. Further studies are required to determine whether the microsporidian presented here belongs to the genus *Heterosporis* or to a polymorphic species group as suggested by the recognition of a robust *Pleistophora/Heterosporis* clade by molecular studies (i.e. possibly representing a cryptic or novel species).

It is becoming increasingly apparent that considerable morphotypic variation occurs not only between, but also within the microsporidians, highlighted by the differential formation of dimorphic spores; the development of monokaryotic or diplokaryotic meronts, sporonts or combinations thereof; the variable persistence of SPV and SPC membranes; the types and arrangements of polar filaments; and the formation of different types of cysts/xenomas. It is plausible that parasite plasticity (recognized amongst microsporidia) and dimorphism amongst class Marinosporidia [17] could explain the possibility of multi-trophic transmission or life-cycle variation of the same taxon occurring within different hosts. Of relevance here is that the manifestations of this morphologic plasticity is generally limited to alterations in karyostatus (monokaryotic vs. diplokaryotic) and cytoplasmic placement [17] rather than variations in exospore arrangement or the number and ranking of polar filaments and, as such, this is unlikely to justify the ultrastructural and molecular paradoxes apparent in this case. In addition, the significance and reliability of the ssrRNA sequence analysis indicating a strong similarity to *Heterosporis anguillarum* needs to be considered. The demonstrated sequence similarity may simply reflect a high degree of genetic conservatism across the microsporidians for at least certain portions of their genome, especially in house-keeping genes with conserved and variable regions. It suggests that examination of other gene sequences may be required to accurately differentiate taxa within this group.

This manuscript outlines in detail the histological, ultrastructural and molecular characteristics of an *Heterosporis*-like microsporidian infecting three Australian sea snake species. Despite in depth analysis of available material, ultrastructural assessment of other development stages would be required to more definitively characterize this organism. This study highlights issues with taxonomic classification of microsporidians based solely on either spore morphology or genomic analysis and demonstrates that sometimes tEM and DNA do not reconcile. Ensuing studies should include detailed assessment of both genotypic as well as morphologic characteristics when attempting to classify microsporidian isolates. This will in turn help to not only expand our understanding of the group, but also reconcile discrepancies within the current taxonomy.

## Supporting Information

S1 Dataset Microsporidia and non-microsporidia sequence alignment. (FAS)
